# Optogenetic induction of contractile ability in immature C2C12 myotubes

**DOI:** 10.1038/srep08317

**Published:** 2015-02-09

**Authors:** Toshifumi Asano, Toru Ishizuka, Keisuke Morishima, Hiromu Yawo

**Affiliations:** 1 Department of Mechanical Engineering, Graduate School of Engineering, Osaka University, 2-1 Yamadaoka, Suita 565-0871, Japan; 2 Japan Science and Technology Agency (JST), Core Research of Evolutional Science & Technology (CREST), 5 Sanbancho, Chiyoda-ku 102-0075, Japan; 3 Department of Developmental Biology and Neuroscience, Tohoku University Graduate School of Life Sciences, 2-1-1 Katahira, Aoba-ku, Sendai 980-8577, Japan; 4 The Center for Advanced Medical Engineering and Informatics, Osaka University, 2-2 Yamadaoka, Suita 565-0871, Japan

## Abstract

Myoblasts can be differentiated into multinucleated myotubes, which provide a well-established and reproducible muscle cell model for skeletal myogenesis *in vitro*. However, under conventional differentiation conditions, each myotube rarely exhibits robust contraction as well as sarcomere arrangement. Here, we applied trains of optical stimulation (OS) to C2C12 myotubes, which were genetically engineered to express a channelrhodopsin variant, channelrhodopsin-green receiver (ChRGR), to investigate whether membrane depolarization facilitates the maturation of myotubes. We found that light pulses induced membrane depolarization and evoked action potentials in ChRGR-expressing myotubes. Regular alignments of sarcomeric proteins were patterned periodically after OS training. In contrast, untrained control myotubes rarely exhibited the striated patterns. OS-trained and untrained myotubes also differed in terms of their resting potential. OS training significantly increased the number of contractile myotubes. Treatment with nifedipine during OS training significantly decreased the fraction of contractile myotubes, whereas tetrodotoxin was less effective. These results suggest that oscillations of membrane potential and intracellular Ca^2+^ accompanied by OS promoted sarcomere assembly and the development of contractility during the myogenic process. These results also suggest that optogenetic techniques could be used to manipulate the activity-dependent process during myogenic development.

Repeated exercise with adequate physical activity is important for health by preventing the development of many chronic diseases such as obesity, type 2 diabetes, sarcopenia, neurodegeneration, and osteoporosis^1^,^2^,^3^. The expression of muscle functionality relies on the correct assembly of myofibrils, which comprise of tandem arrays of minimum contractile units, i.e., sarcomeres. Muscle activity such as excitation and contraction is known to regulate the expression and modification of a number of molecules such as acetylcholinesterase^4^, L-type Ca^2+^ channels^5^, and glucose transporters^5^,^6^. Previously, it was reported that muscle fiber stimulation with electrical^7^,^8^,^9^, mechanical^10^,^11^, or pharmacological^12^,^13^ methods, which mimic motor neuron inputs, facilitate the maturation of developing muscles as well as the maintenance of contractility. Cultured myogenic cells subjected to electrical stimuli with a given temporal pattern *in vitro* exhibited accelerated sarcomere assembly, and the contractile ability was induced by facilitating the production of elongation factors and muscle proteins^14^,^15^. Electrical field stimulation using electrodes placed in the extracellular space is a simple method for controlling the temporal pattern of muscle activation. However, it is difficult to identify the activated cells because the electric field is generally widespread and nonuniform. In addition, the placement of metal electrodes in the extracellular space has invasive effects on cells because they generate toxic gases such as H_2_ and Cl_2_ and change pH due to a Faradaic reaction. Therefore, the magnitude and duration of electrical field is limited within the range of a few volts/mm and milliseconds to minimize the side effects. However, optogenetic approaches using light-sensitive ion channels, channelrhodopsins (ChRs), have attracted attention as a new method for stimulating cells and tissues^16^,^17^,^18^,^19^, and their application is expanding in the field of neuroscience^20^,^21^,^22^,^23^. We have previously demonstrated that muscular contraction was optically controlled in a synchronous manner using a given train of light pulses when a myotube was generated from C2C12 clonal myoblasts, which were genetically engineered to express channelrhodopsin-2 (ChR2)^24^. However, previous studies have not demonstrated whether it is possible to manipulate the developmental process in any biological tissues^25^.

Thus, we hypothesized that oscillatory changes of membrane potential after manipulation by optogenetic stimulation would induce the morphological and functional acquisition of the contractile ability in immature muscle. To test this hypothesis, optical stimulation (OS) training was applied to the ChR-expressing myotubes derived from C2C12, a myoblast cell line. We found that flashing a light with a specific rhythm facilitated both sarcomere assembly and the development of contractility in the ChR-expressing myotubes. Our results suggest that the morphological maturation and functional development of C2C12 myotubes is accelerated by rhythmic changes of membrane potential, which is manipulated by light using an optogenetic technique.

## Results and Discussion

### Formation of the ChR-expressing myotubes

**Figure 1:**
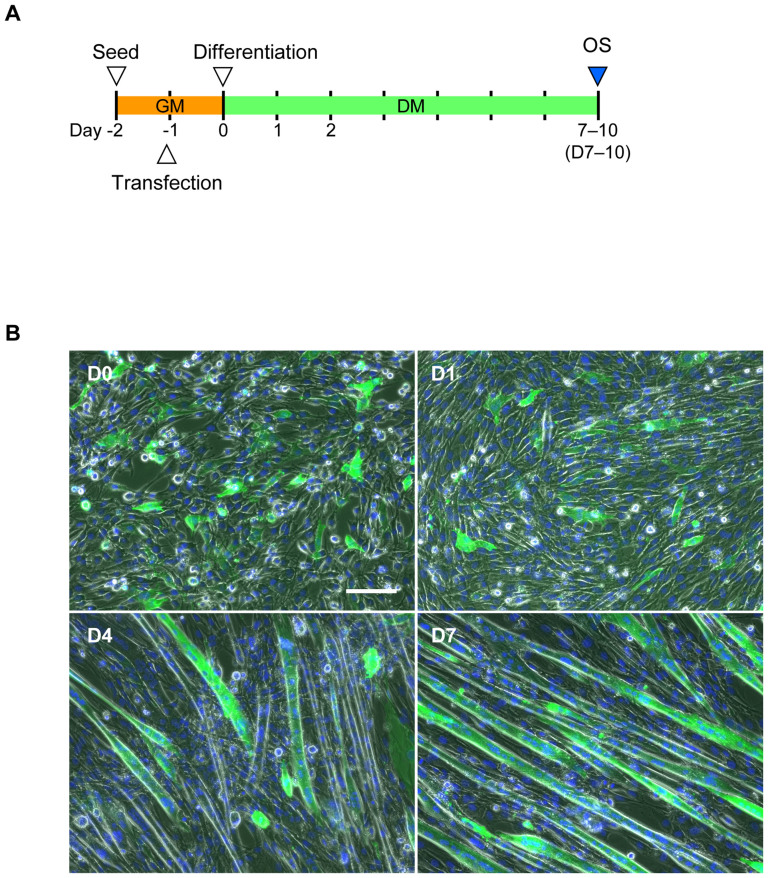
Generation of photosensitive C2C12 myotubes. (A) Time schedule for the OS training assay with C2C12 myotubes. One day after seeding (Day −1), cells were transfected with ChRGR-Ve in the growth medium (GM). Twenty-four hours later (Day 0, D0), the medium was replaced with differentiation medium (DM) to promote the formation of multinuclear myotubes. The OS training assay was performed on Days 7–10. (B) Generation of ChRGR-Ve-expressing multinucleated myotubes on day 0 (D0), day 1 (D1), day 4 (D4), and day 7 (D7) after switching to DM. Multinucleated myotubes expressing ChRGR were detected based on Venus-fluorescence (green), and the cell nuclei were stained with Hoechst 33258 (blue). Scale bar, 50 μm.

To apply optogenetics, we selected ChR-green receiver (ChRGR), a chimera of ChR1 and 2, because^26^ it evokes a relatively large photocurrent with minimal desensitization, it is expressed efficiently in the membrane, and it exhibits rapid photocurrent kinetics in both on-off states and during recovery from desensitization. Twenty-four hours after transfection with ChRGR (Fig. 1A; Day 0, D0), some C2C12 myoblasts were found to express ChRGR with bright Venus fluorescence (ChRGR-Ve), whereas others exhibited no fluorescence (Fig. 1B, D0). Subsequently, the mixture of ChRGR-Ve-positive and -negative C2C12 cells was allowed to differentiate in standard differentiation conditions^27^,^28^. The C2C12 cells, either fluorescent or non-fluorescent, began to fuse each other to form multinucleated myotubes^29^,^30^ after several days (Fig. 1B, D1–D7). Some of these multinucleated myotubes expressed ChRGR-Ve, whereas others did not. The ChRGR-Ve-positive myotubes were easily discriminated from the non-fluorescent ones and they comprised approximately 50%–60% of the multinucleated myotubes at D7, although the expression level was variable.

### Light sensitivity of multinucleated myotubes

**Figure 2:**
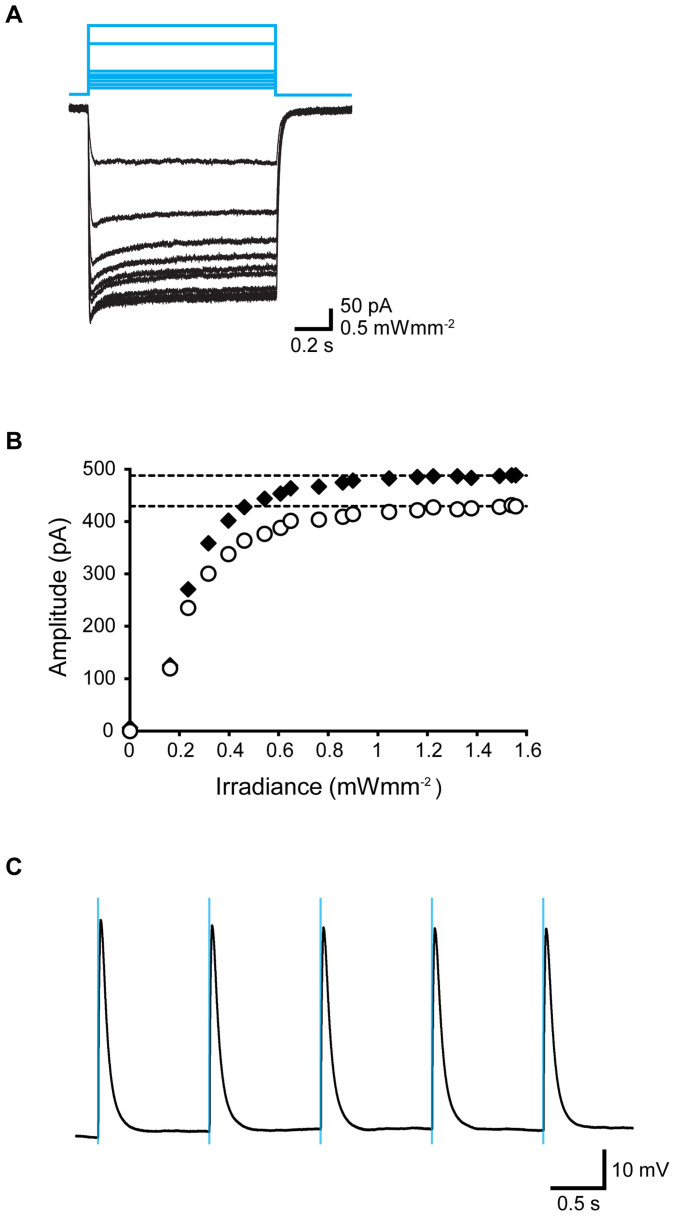
Optical responses of ChRGR-expressing C2C12 myotubes. (A) Typical photocurrent traces (black lines) from a photosensitive multinucleated myotube induced by cyan LED light at various intensities (blue lines; 0.16–1.58 mWmm^−2^). (B) Power dependency of the peak current (filled diamonds) and steady-state current amplitudes (open circles). (C) Rhythmic action potentials generated by repetitive cyan LED flashes (blue lines; duration, 20 ms; frequency, 1 Hz) with the current clamp.

The photosensitivity of fluorescent multinucleated myotubes was investigated using the whole-cell patch-clamp method at −60 mV. Irradiation with a cyan LED light evoked an inward photocurrent in a power-dependent manner (Fig. 2A). The photocurrent consisted of a peak and a steady state with minimal desensitization, as reported previously^26^. Each photocurrent, peak, or steady-state reached its maximum approximately at 0.8–0.9 mWmm^−2^ of cyan LED light (Fig. 2B). In the current-clamp mode, pulsative irradiation (duration, 20 ms) of cyan LED light induced rapid membrane depolarization and it evoked an action potential, which was robustly phase-locked to the flashing light (Fig. 2C). The same irradiation produced neither photocurrent nor depolarization in nonfluorescent cells, which was consistent with previous studies^31^,^32^.

### Sarcomere assembly by OS training

**Figure 3:**
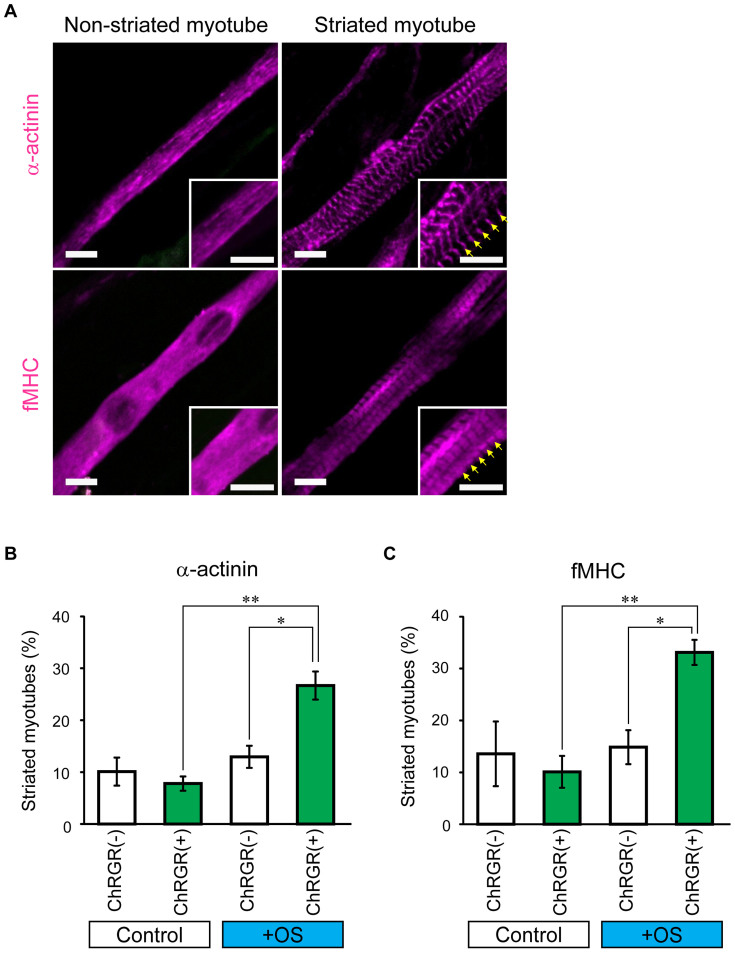
Effects of OS training on sarcomere assembly in ChRGR-expressing C2C12 myotubes. (A) Images of typical multinucleated myotubes 7 days differentiation induction. Cells were fixed and stained with anti-sarcomeric α-actinin (upper panel) or fMHC (lower panel). The insets show the magnified images. The sarcomeric structures are indicated by arrows. Scale bar, 10 μm. (B) Fractions of myotubes with mature striation patterns. Assembly of α-actinin (from left to right): control ChRGR-negative myotubes without OS training (white column, n = 12 regions), ChRGR-positive myotubes without OS training (green column, n = 12 regions), ChRGR-negative myotubes with OS training (white column, n = 16 regions), and ChRGR-positive myotubes with OS training (green column, n = 16 regions). (C) Similar to (B), but showing the assembly of fMHC in ChRGR-negative (white bar, each, n = 16 regions) and –positive cells (green bar, each, n = 16 regions). Each experiment consists of at least three independent replicates. *, *P* < 0.05; **, *P* < 0.01; Kruskal–Wallis test.

Under conventional myogenic culture conditions, C2C12 myotubes generally remained immature with minimal development of sarcomeric structure and contractility^27^,^33^. Extracellular electrical stimulation of multinucleated myotubes has been reported to accelerate the organization of sarcomeric proteins to assemble to form sarcomeres, which are the smallest contractile units in muscle^8^,^15^. We hypothesized that a certain OS pattern would facilitate sarcomere assembly in ChRGR-Ve-positive myotubes. To test this hypothesis, the contribution of OS training was evaluated based on the distributions of the Z-line protein, α-actinin, and the A-band protein, skeletal fast myosin heavy chain (fMHC), shortly after OS training. The cyan LED light was irradiated in a certain pattern as the OS training with the irradiance larger than 0.67 mWmm^−2^, which generated a near maximal photocurrent (Fig. 2B). The ChRGR-Ve-positive or negative myotubes generally exhibited a nonstriated morphology before OS training (Supplementary Figure S1A). Thus, both α-actinin and fMHC were distributed with punctate patterns throughout the cytoplasm or in a continuous pattern along filamentous structures in the myotubes (Fig. 3A). On the other hand, the mature striation pattern of α-actinin and fMHC appeared after the 1-Hz OS training protocol (Fig. 3A, arrows and Supplementary Figure S1B). Because the striations of α-actinin and fMHC were both aligned regularly along the longitudinal axis of the multinucleated myotubes, they were organized to form sarcomeric structures that possessed the Z-line and A-band. The fraction of ChRGR-Ve-positive myotubes that were striated with α-actinin was 27 ± 10% (range, 7.7%–44%, n = 16 regions) after OS training (1 Hz). This was significantly larger than that of nonfluorescent myotubes in the same regions as well as that of either fluorescent or nonfluorescent myotubes at the same stage without OS (Fig. 3B). Similarly, the fraction of ChRGR-Ve-positive myotubes that were striated with fMHC was 33 ± 9.6% (range, 19%–50%, n = 16 regions) after OS training (1 Hz), which was significantly larger than that of nonfluorescent myotubes in the same regions, as well as that of fluorescent or nonfluorescent myotubes at the same stage without OS training (Fig. 3C). These results suggest that OS training induced efficient sarcomere assembly specifically in ChRGR-expressing myotubes.

### Membrane properties after OS training

**Figure 4:**
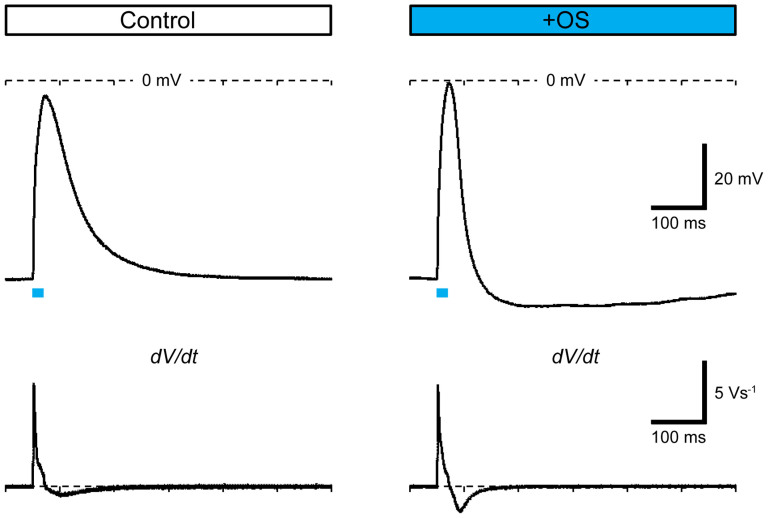
Electrical properties of membranes. Typical action potentials (upper traces) and their time-derivatives (*dV/dt* data, lower traces) generated by cyan LED flashes (20 ms, indicated by blue lines) in ChRGR-expressing myotubes without (left) or with OS training (right).

C2C12 myotubes also have immature membrane properties such as small amplitudes in E_rest_ and V_AP_. Therefore, we investigated the electrophysiological properties of the C2C12 myotube membranes using the whole-cell patch-clamp method after the continuous application of OS training at 1 Hz for 2 h. The OS training protocol improved the membrane properties of fluorescent myotubes to some extent (Table 1). R_in_ and C_cell_ remained relatively unchanged compared with that of control myotubes. In OS-trained myotubes, E_rest_ was −42.6 ± 3.18 mV (range = −60 to −33 mV, n = 8 cells), which was significantly more negative than that in the untrained myotubes (range = −39 to −21 mV, n = 10 cells), and this was consistent with previous studies^34^. The active membrane properties were examined while holding the resting potential at −60 mV by injecting DC current (Fig. 4). However, OS training did not improve the amplitude of V_AP_ or [*dV/dt*]_max_, which is an indicator of Na^+^ channel activity. It has a tendency to increase the negativity of [*dV/dt*]_min_, which is an indicator of K^+^ channel activity, from −0.95 ± 0.18 V/s (range = −0.12 to −1.92 V/s, n = 10 cells) to −1.32 ± 0.29 V/s (range = −0.30 to −2.66 V/s, n = 8 cells) with insignificant difference (Table 1). These results suggest that OS training minimally influence the active membrane properties. There is also no evidence that OS training enhanced the development of the tubular membrane system because it did not affect R_in_ or C_cell_.

### Induction of the contractile ability by OS training

**Figure 5:**
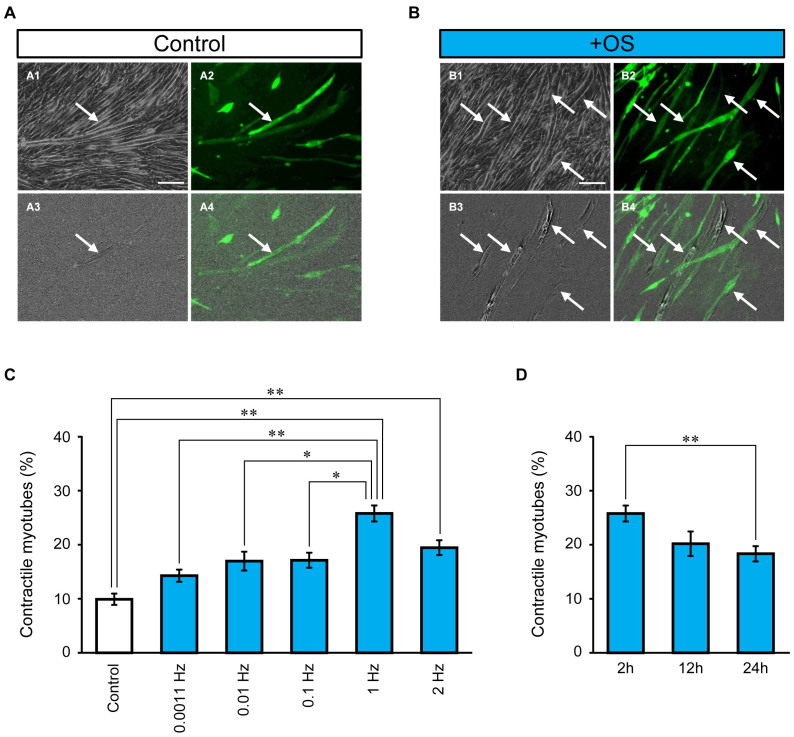
Induction of contractile ability. (A) Image difference extraction analysis of myotubes without OS training: phase-contrast image (A1), ChRGR-Ve-fluorescence image (A2), difference image (A3), and merged image of A2 and A3 (A4). Contracted myotubes are indicated by arrows. (B) Myotubes after OS training. Scale bar, 100 μm. (C) Effects of OS protocols on the fraction of contractile fluorescent myotubes divided by the total fluorescent myotubes (from left to right): control without OS (n = 24 regions), 0.0011 Hz (n = 20 regions), 0.01 Hz (n = 20 regions), 0.1 Hz (n = 28 regions), 1 Hz (n = 32 regions), and 2 Hz (n = 16 regions). (D) Fraction of contractile myotubes with OS protocols at 1 Hz for (from left to right): 2 h (n = 32 regions), 12 h (n = 12 regions), and 24 h (n = 16 regions). Each experiment consists of at least four independent replicates. *, *P* < 0.05; **, *P* < 0.01; Kruskal–Wallis test.

In general, C2C12 myotubes hardly exhibit the robust contraction spontaneously under conventional culture conditions. It is likely that this contractility depends on the assembly of sarcomeres in multinucleated myotubes^8^,^9^,^15^. To investigate the effect of OS training on the induction of the contractile ability, OS training was applied to C2C12 myotubes using the 1 Hz protocol. The control groups without OS training were unresponsive to the cyan LED flash at any frequency or intensity (Fig. 5A and Supplementary Movie S1). In contrast, contractions appeared in fluorescent myotubes in response to a cyan LED flash after OS training (Fig. 5B and Supplementary Movie S2). The fraction of contractile myotubes comprised 26 ± 8.4% (range = 9.5%–50%, n = 32 regions) of ChRGR-Ve-positive myotubes trained by 1 Hz OS-protocol (Fig. 5C), which was significantly larger than that without OS training (9.9 ± 5.1%; range = 0%–21%, n = 32 regions). The induction of the contractile ability also depended on the OS protocol, which was applied for 2 h. Low-frequency protocols such as 0.0011, 0.01, and 0.1 Hz were significantly less effective than the standard protocol of 1 Hz. However, the 2 Hz protocol was less effective than the standard protocol. The effectiveness of OS training also depended on the treatment time (Fig. 5D) where longer OS protocols such as 1 Hz for 24 h were significantly less effective than the standard protocol (1 Hz for 2 h). Based on these results, we concluded that rhythmical OS at 1 Hz for 2 h was suitable for developing contractility in ChRGR-Ve-positive multinucleated myotubes and it was used as the standard.

### Involvement of intracellular Ca^2+^

**Figure 6:**
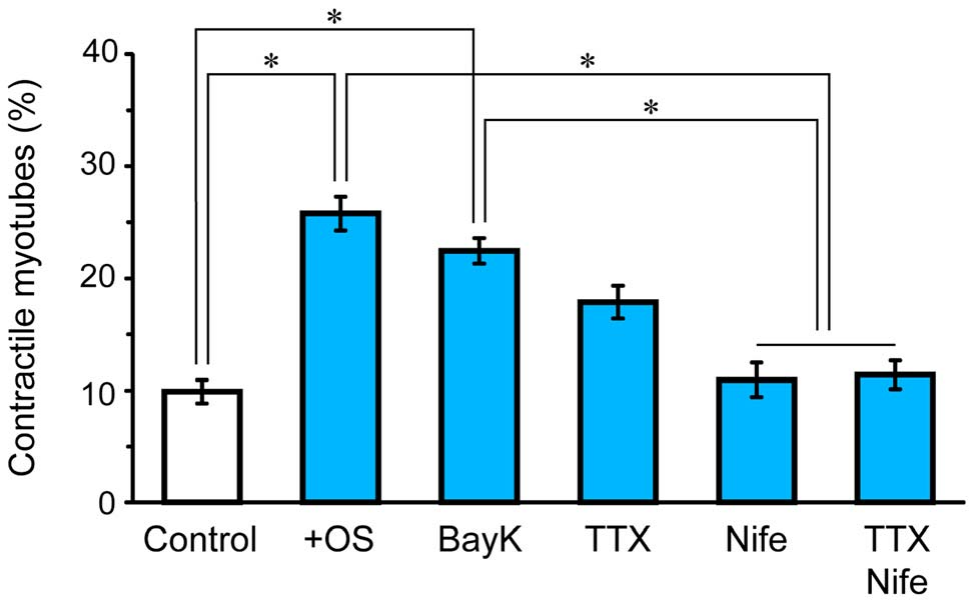
Effects of pharmacological treatments on the induction of the contractile ability. From left to right: without OS training as a reference (n = 24 regions), OS training without any treatment (n = 32 regions), BayK (n = 20 regions), TTX (n = 20 regions), Nife (n = 24 regions), and TTX and Nife (n = 16 regions) in ChRGR-expressing myotubes. Each experiment consists of at least four independent replicates. *, *P* < 0.01; Kruskal–Wallis test.

It is hypothesized that the OS training protocol induced a rhythmic change of membrane potential and the subsequent intracellular Ca^2+^ oscillation, which contributed to the induction of the contractile ability. To test this hypothesis, we examined the effect of various pharmacological treatments on the acquisition of the contractile ability. Bay K 8644 (BayK; an agonist of L-type Ca^2+^ channels, LTCCs), nifedipine (Nife; an antagonist of LTCCs and excitation-contraction (EC) coupling), and tetrodotoxin (TTX, a blocker of voltage-dependent Na^+^ channels) were added to the medium during OS training in separate trials. As summarized in Fig. 6, BayK or TTX had no significant effects on the fraction of contractile myotubes, whereas Nife reduced the fraction to the level without training. These results suggest that the rhythmical oscillation of membrane potential and the accompanying intracellular Ca^2+^ changes may facilitate the development of contractility.

In skeletal muscle cells, the action potential evoked by membrane depolarization is generally conducted along the surface membrane into an invaginated tubular system network. This in turn activates LTCCs/dihydropyridine receptors (DHPRs), which undergoes conformational changes to allow Ca^2+^ release from ryanodine receptors (RyRs) in the sarcoplasmic reticulum (SR)^35^,^36^. At present, we do not have any evidences that the ChRGR-expressing myotubes either develop the tubular system, as previously reported^37^, or the EC coupling molecular complex. However, we could presume that the whole plasma membrane of a ChRGR-expressing myotube would be depolarized at once directly by light. The lower sensitivity to TTX occurred because the LTCCs/DHPRs were activated directly by light-induced membrane depolarization but without the propagation of action potentials. This increased the intracellular Ca^2+^ level by Ca^2+^ influx and/or via RyRs. The rhythmic oscillation of intracellular Ca^2+^ subsequently facilitated sarcomere assembly. Thus, it is possible that sarcomere assembly may be a prerequisite for light-evoked contraction.

Thus, in this study, we demonstrated that OS training using a flash light with a rhythmical frequency facilitated sarcomere assembly to form the specific structural alignment of sarcomeric proteins, which are involved in muscle contraction. OS training also induced the contractile ability in C2C12 myotubes, which were genetically engineered to express ChRGR. Our results suggest that optogenetic techniques are useful for manipulating the morphological maturation and functional development of C2C12 myotubes.

## Methods

### Cell cultures and transfection

C2C12 cells are a myoblast line derived from mouse satellite cells (RIKEN Cell Bank), which have been used as models in studies of skeletal muscle development. The cells were maintained for no more than six passages at 37°C with a 5% CO_2_ atmosphere in Dulbecco's Modified Eagle's Medium (DMEM, Wako Pure Chemical Industries, Osaka, Japan), which was supplemented with 10% fetal bovine serum (Invitrogen, Carlsbad, CA), 100 units/mL penicillin, and 100 μg/mL streptomycin (Sigma-Aldrich, St Louis, MO). Fragments of cDNA that encoded the codon-humanized ChRGR-Venus conjugate (ChRGR-Ve) were amplified by PCR and subcloned into EcoRI sites in pCAGGS by In-Fusion cloning (Takara Bio, Shiga, Japan), as described previously^38^. C2C12 myoblasts were grown to 80%–90% confluence on 35 mm-diameter tissue culture dishes coated with rat tail collagen and subsequently transfected with ChRGR-Ve expression vector plasmids using Lipofectamine 2000 transfection reagent (Invitrogen), according to the manufacturer's instructions. Twenty-four hours post-transfection (Day 0), differentiation into multinuclear myotubes was facilitated by switching to differentiation medium (DM), i.e., DMEM containing 2% horse serum (Invitrogen), 1 nM insulin (Invitrogen), 100 units/mL penicillin, 100 μg/mL streptomycin, 200 μM L-ascorbic acid, and Mg-phosphate salt n-hydrate (Wako Pure Chemical Industries). The DM was replenished every day.

### OS training

To perform OS training, we used a custom-built irradiation system, which consisted of an array of seven LEDs arranged in series on a board (281MCPCB, Polymer Optics Ltd., England). Cyan LEDs (505 ± 15 nm, LXML-PE01-0070, Philips Lumileds Lighting Co., San Jose, CA) were regulated using a pulse generator (SEN-7203, Nihon Kohden, Tokyo, Japan) with a current booster (SEG-3104, Nihon Kohden). The power density of the LED arrays through a diffuser lens (Polymer Optics Ltd) was ≧ 0.67 mWmm^−2^ at the bottom of the culture dish, which was measured directly using a visible light-sensitive thermopile (MIR-100Q, Mitsubishi Oil Chemicals, Tokyo, Japan). OS training was conducted with C2C12 myotubes for 7–10 days (D7–10) after switching to DM. During the application of an OS training protocol for 2 h at 37°C in a 5% CO_2_ atmosphere, the medium was replaced temporarily with OS medium, which consisted of phenol red-free DMEM (Invitrogen) supplemented with 2% horse serum, 2% MEM amino acids solution (Invitrogen), 1% MEM nonessential amino acids solution (Invitrogen), 100 units/mL penicillin, and 100 μg/μL streptomycin. The stimulation protocols were applied at the following frequencies: 0.0011 Hz (duration, 18 s; 8 pulses), 0.01 Hz (duration, 2 s; 72 pulses), 0.1 Hz (duration, 200 ms; 720 pulses), 1 Hz (duration, 20 ms; 7,200 pulses), and 2 Hz (duration, 10 ms; 14,400 pulses). In each protocol, the total irradiation time was 144 s in 2 h. In the control experiments, the C2C12 myotubes were exposed to the same OS medium as the stimulation groups but without any OS training protocols.

### Electrophysiology

ChRGR-Ve-positive myotubes were identified by Venus-fluorescence using a conventional epifluorescence microscope (BX51WI, Olympus, Tokyo, Japan), which was equipped with a 60× water-immersion objective lens (LUMplanPl/IR60x, Olympus) and a filter cube (excitation, 495 nm; dichroic mirror, 505 nm; barrier filter, 515 nm). Electrophysiological recordings were performed in the whole-cell patch-clamp mode using a conventional system (Axopatch 200A plus Digidata1200, Molecular Devices, Sunnyvale, CA) and with pCLAMP 10.2 computer software (Molecular Devices). The standard extracellular Tyrode's solution contained (in mM): 138 NaCl, 3 KCl, 2.5 CaCl_2_, 1.25 MgCl_2_, 10 HEPES, 4 NaOH, and 11 glucose (pH 7.4 adjusted with HCl). The standard patch pipette solution contained (in mM): 120 CsOH, 100 glutamic acid, 0.2 EGTA, 10 HEPES, 2.5 MgCl_2_, 3 MgATP, 0.3 Na_2_GTP, and 0.1 leupeptin (pH 7.4 adjusted with CsOH) for the voltage clamp, or 125 K-gluconate, 10 KCl, 0.2 EGTA, 10 HEPES, 1 MgCl_2_, 3 MgATP, 0.3 Na_2_GTP, and 0.1 leupeptin (pH 7.4 adjusted with KOH) for the current clamp. To test the optical responses, we used a cyan LED (505 ± 15 nm, LXHL-NE98, Philips Lumileds Lighting Co.), which was regulated by a pulse generator (SEN-7203) and pCLAMP 10.2 computer software. The maximum power density of the LED light through an objective lens was 1.58 mWmm^−2^ at the point of focus on the specimen. The photocurrent response induced by the LED light was recorded using a CsCl-based internal solution with the whole-cell voltage clamp. The electrophysiological properties were characterized using a K-gluconate-based internal solution to determine the input resistance (R_in_), whole cell capacitance (C_cell_), resting membrane potential (E_rest_). R_in_ and C_cell_ were calculated from a current response to the 50 ms, 10 mV hyperpolarizing pulse. To evaluate the cell's excitability, the membrane potential responses to the light pulses (20 ms) were recorded while holding the E_rest_ at −60 mV under current clamp. They were digitally time-differentiated and digitally smoothed by a low-pass filter at 200 Hz (*dV/dt* data), and action potential amplitude (V_AP_), the maximal and the minimal value of *dV/dt* data, [*dV/dt*]_max_ and [*dV/dt*]_min_ were characterized. All of the raw data were filtered at 2 kHz and sampled at 10 kHz. All of the experiments were performed at room temperature.

### Immunohistochemistry

Cells were fixed with 4% paraformaldehyde/PBS immediately after the OS training protocol and permeabilized with 0.1% Triton X-100/PBS for 15 min. The control myotubes without OS training were processed in a similar manner at the same stage. The samples were blocked in PBS containing 5% goat serum for 1 h and treated with the primary antibodies, i.e., rat monoclonal anti-GFP IgG2a (1:1000, GF090R, Nacalai Tesque, Kyoto, Japan), mouse monoclonal anti-sarcomeric α-actinin IgG1 (1:500, EA-53, Sigma-Aldrich), and mouse monoclonal anti-myosin heavy chain IgG1 (fMHC; 1:500, MY-32, Sigma-Aldrich), diluted in PBS containing 1% goat serum for 1 h, and then they were incubated for 1 h with the secondary antibodies, i.e., Alexa Fluor 488-conjugated goat anti-rat IgG (Invitrogen) and Alexa Fluor 546-conjugated goat anti-mouse IgG (Invitrogen), diluted at 1:200 in PBS containing 1% goat serum. The samples were washed three times with PBS between treatments. The specimens were mounted with Vectashield (Vector Laboratories, Burlingame, CA) and coverslipped. All of the above reactions were performed at room temperature. The fluorescent images were captured with a conventional confocal laser microscope (LSM510META, Carl Zeiss, Oberkochen, Germany), which was equipped with a 40× water immersion objective and corrected for brightness and contrast using conventional software (LSM Image Browser).

### Analysis of contractility

The contractility of myotubes was monitored sequentially using a CCD camera (VB-7010, Keyence Co., Osaka, Japan; 7.5 frames/s) by phase contrast/epifluorescence microscopy with a 20× objective lens and a GFP filter set (excitation, 485 nm; dichroic mirror, 510 nm; bandpass filter, 515–565 nm) to observe Ve-fluorescence. The myotubes were photostimulated using a cyan LED flash (peak, 490–520 nm; 0.55 mWmm^−2^ at the focus; 1 Hz for 100 ms) through the objective lens with a filter set (dichroic mirror, 550 nm; long pass filter, 565 nm), which was regulated by a pulse generator (SEN-7203). Contractile myotubes were identified using image difference extraction analysis; the phase-contrast image before a cyan LED flash was digitally subtracted from the image immediately after the flash and overlaid on the fluorescence image. Thus, the difference extraction images indicated the contractility of fluorescent myotubes. Each 35 mm dish was divided into four regions and images were recorded from several visual fields unintentionally located in each region. The numbers of fluorescent myotubes and contractile fluorescent myotubes were then counted. The fraction of contractile myotubes was calculated as the total number of contractile fluorescent myotubes divided by the total fluorescent myotubes in each region. All of the image analysis was performed using ImageJ software (NIH, Bethesda, MD).

### Pharmacological treatments

In the pharmacological experiments, 0.3 μM BayK ((*S*)-(-)-Bay K 8644, Sigma-Aldrich), an agonist of L-type Ca^2+^ channels (LTCCs), 2 μM TTX (Tocris Bioscience, Ellisville, MO), a blocker of voltage-gated Na^+^ channels, 10 μM Nife (Sigma-Aldrich), an antagonist of LTCCs and an inhibitor of EC coupling were added to the medium at these final concentrations. The cells were incubated in OS medium containing each reagent for at least 15 min before the stimulation protocol. During OS training (20 ms duration, 1 Hz frequency) for 2 h, cell detachment and morphological changes were not observed in these conditions. After OS training with pharmacological treatments, myotubes were washed five times with Tyrode's solution and incubated for 5 min, and the contractility was evaluated as described previously.

### Statistical analysis

All data in the text and figures were expressed as the mean ± SEM and evaluated using one-way Kruskal–Wallis and Mann–Whitney U–test to determine statistically significant differences, unless stated otherwise. *P* < 0.05 indicated a statistically significant difference.

## Supplementary Material

Supplementary Information Supplementary information

Supplementary Information Supplementary Movie S1

Supplementary Information Supplementary Movie S2

## Figures and Tables

**Table 1:** Summary of the electrophysiological properties of the membranes of ChRGR-expressing C2C12 myotubes without (control) or with OS training

	R_in_ (MΩ)	C_cell_ (pF)	E_rest_ (mV)	V_AP_ (mV)	[*dV/dt*]_max_ (Vs^−1^)	[*dV/dt*]_min_ (Vs^−1^)
Control (n = 10)	131 ± 18.3	383 ± 61.1	−32.5 ± 1.74	44.2 ± 6.29	6.11 ± 1.36	−0.95 ± 0.18
OS training (n = 8)	131 ± 28.5	406 ± 74.1	−42.0 ± 2.81*	45.7 ± 5.40	6.93 ± 1.55	−1.32 ± 0.29

The values represent the mean ± S.E.M. (n = 8–10). *, *P* < 0.05; Mann–Whitney *U*–test.
