# Supplementary material for: Optogenetic induction of contractile ability in immature C2C12 myotubes
Source: Sci Rep. 2015 Feb 9;5:8317. doi: 10.1038/srep08317 (PMC4650824; doi:10.1038/srep08317)

Supplementary Information

Optogenetic induction of contractile ability in immature C2C12 myotubes

Toshifumi Asano[1,2 *], Toru Ishizuka[2,3], Keisuke Morishima[1,4], and Hiromu Yawo[2,3]

[1]Department of Mechanical Engineering, Graduate School of Engineering, Osaka University, 2-1 Yamadaoka, Suita 565-0871, Japan

[2]Japan Science and Technology Agency (JST), Core Research of Evolutional Science & Technology (CREST), 5 Sanbancho, Chiyoda-ku 102-0075, Japan

[3]Department of Developmental Biology and Neuroscience, Tohoku University Graduate School of Life Sciences, 2-1-1 Katahira, Aoba-ku, Sendai 980-8577, Japan

[4]The Center for Advanced Medical Engineering and Informatics, Osaka University, 2-2 Yamadaoka, Suita 565-0871, Japan

*Correspondence to asano.cbio@tmd.ac.jp

Supplementary Figure S1

Sarcomere assembly with optical stimulation (OS) training. (A) A typical unstimulated channelrhodopsin-green receiver (ChRGR)-expressing myotube (control) and (B) another typical myotube immediately after OS training at 1 Hz for 2 h. Each image shows the immunohistochemical reaction to (from left to right) anti-GFP (green), anti-sarcomeric α-actinin or fMHC (magenta), and a merged image of both. The insets are magnified images. The sarcomeric structures are indicated by arrows. Scale bar, 10 μm.

Supplementary Movie S1

Untrained responses of C2C12 myotubes to light pulses (duration, 100 ms; frequency, 1 Hz). The fluorescence image was overlaid on the phase-contrast image.

Supplementary Movie S2

Responses of optical stimulation (OS)-trained C2C12 myotubes to light pulses (duration, 100 ms; frequency, 1 Hz). The contractile myotubes were all fluorescent with variable intensities.

Supplementary Figure S1

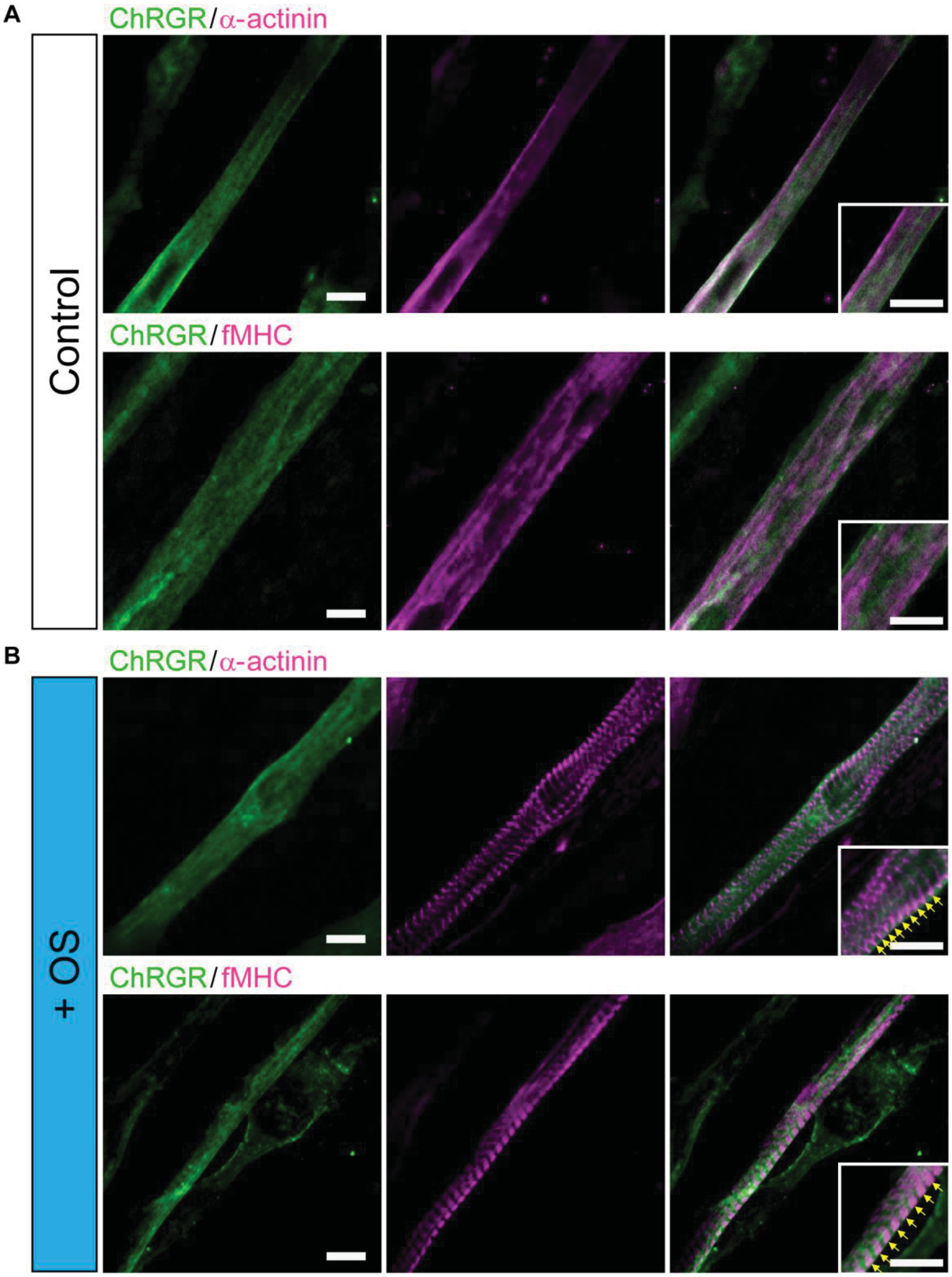

Supplement: Supplementary Information [file srep08317-s1.pdf]
